# Outpatient Safety for Pediatric Patients With Severe Obstructive Sleep Apnea Undergoing Adenotonsillectomy

**DOI:** 10.7759/cureus.114208

**Published:** 2026-08-09

**Authors:** Natalie M Wall, Theresa A Schneider, Nathaniel G Blanchard, Prasad J Thottam, Colin T Bohr, Michael S Haupert

**Affiliations:** 1 Otolaryngology - Head and Neck Surgery, Henry Ford Warren Hospital, Warren, USA; 2 Medicine, Michigan State University College of Osteopathic Medicine, East Lansing, USA; 3 Otolaryngology - Head and Neck Surgery, Michigan Pediatric ENT Associates, West Bloomfield, USA

**Keywords:** clinical guidelines, pediatric surgery, postoperative admissions, postoperative complications, sleep surgery

## Abstract

Objective

The objective of this study is to evaluate the admission guidelines and safety of outpatient tonsillectomy and adenoidectomy (T&A) for pediatric patients with severe obstructive sleep apnea (OSA).

Methods

A retrospective chart review was performed by electronic medical record review. Eighty-seven pediatric patients (aged 3-17) were included; all underwent a T&A at a pediatric tertiary care center. All patients underwent polysomnography (PSG) to diagnose the presence and severity of OSA. After obtaining PSG results, these patients underwent T&A. The control group included admitted patients with an apnea-hypopnea index (AHI) of 10-20/hr, and the treatment group included discharged patients with an AHI of 10-20/hr. For the patients admitted, any oxygen desaturations overnight or oxygen supplementation required were documented. For all patients, return to the Emergency Department (ED) or an Urgent Care (UC) after discharge from the procedure was documented. A statistical analysis was then completed on the data.

Results

No respiratory-related events were documented for patients with an AHI less than 20/hr during overnight observation after T&A. Of the control group, only two were noted to have a desaturation event. None of the patients in the control group required supplemental oxygen overnight. Only one patient in this study required supplemental oxygen after a desaturation event, and their preoperative AHI was 46/hr. Three patients from the control group returned to the ED after discharge, and only two patients from the treatment group returned to the ED after discharge. However, none of the treatment group patients returned to the ED due to a respiratory-related event.

Conclusion

Current clinical practice guidelines in the United States recommend admission for pediatric patients with an AHI of ≥10 events per hour after T&A for OSA; however, this study provides support for the safe discharge of patients with an AHI less than 20 events per hour. This adjustment has the potential to decrease unnecessary hospital admissions, lower associated healthcare costs, and reduce the incidence of hospital-acquired complications. Given the retrospective, single-center cohort design of this study, larger multicenter prospective studies are warranted to further evaluate and validate these findings before changes to current clinical practice guidelines are considered.

## Introduction

Tonsillectomy, with or without adenoidectomy, is among the most frequently performed surgical procedures in the pediatric population. In the United States, approximately 530,000 tonsillectomies are performed annually in children under 15 years of age [1]. This procedure entails the complete excision of tonsillar tissue and its capsule from the pharyngeal musculature and is primarily indicated for the management of obstructive sleep apnea (OSA) and recurrent tonsillitis [1].

OSA represents a common indication for tonsillectomy and adenoidectomy (T&A) due to its association with upper airway obstruction during sleep. Symptoms of OSA include snoring, mouth breathing, witnessed apneas, restless sleep, irritability, daytime sleepiness, and nocturnal enuresis [2]. Polysomnography (PSG) is routinely employed as part of the diagnostic evaluation to quantify the severity of sleep-disordered breathing. A central metric derived from PSG is the apnea-hypopnea index (AHI), which measures the number of apneic and hypopneic events per hour of sleep. AHI values for pediatric patients are conventionally classified as mild (one to less than five events per hour), moderate (five to less than ten events per hour), or severe (≥10 events per hour) [3]. Elevated AHI scores have been correlated with increased postoperative risk.

However, despite the apparent utility of AHI in predicting postoperative outcomes, there is currently no definitive global threshold used to determine ideal postoperative disposition in pediatric patients undergoing adenotonsillectomy, and therefore institutional practices appear to vary regarding the application and interpretation of AHI values.

In terms of current practice guidelines, the American Academy of Otolaryngology-Head and Neck Surgery Foundation (AAO-HNSF), updated in 2019, recommends inpatient monitoring for children under three years of age or for those with an AHI ≥10/hr, an oxygen saturation nadir below 80%, or both [4]. Accordingly, many institutions routinely admit pediatric patients with pre-operative AHI scores ≥10/hr for overnight observation, irrespective of intraoperative findings or postoperative clinical status [5].

Given the imperative to enhance pediatric patient care, it is critical to re-evaluate widely adopted guidelines that inform clinical decision-making. This study aims to assess whether the current AHI threshold can be modified to a higher threshold, while still ensuring safe patient outcomes. If such a revision leads to reduced utilization of limited hospital resources, without affecting patient safety, it could offer significant benefits to the healthcare system. Optimizing this threshold has the potential to decrease unnecessary hospital admissions, lower associated healthcare costs, and reduce the incidence of hospital-acquired complications.

## Materials and methods

Study design and participants

This retrospective review was performed on patients who underwent T&A from January 2022 to June 2024 at a tertiary care pediatric hospital (Corewell Royal Oak Hospital, Royal Oak, USA). A total of 87 patients were included in this study. All study protocols were approved by the study hospital's Institutional Review Board (IRB - Corewell Royal Oak 2025-002). Inclusion criteria were patients aged three to 18 years diagnosed with OSA. Exclusion criteria included patients without a PSG, patients less than three years old, and pregnancy. 

Polysomnography sleep study

All children involved underwent PSG to diagnose the presence of OSA and stratify the severity. OSA severity was defined based on AHI per practice guidelines: mild was one to less than five events per hour, moderate was five to less than 10 events per hour, and severe was ≥10 events per hour. 

Admission versus discharge

Preoperative AHI was used to determine whether a patient was admitted or discharged. For the control group, patients with an AHI >10/hr were admitted, and those with an AHI <10/hr were discharged. For the treatment group, patients with an AHI <20/hr were discharged, while patients with an AHI score ≥20/hr were admitted. The determination of admission versus discharge was at the surgeon's preference. Three pediatric otolaryngology fellowship-trained physicians performed the procedures in this study. Patients who were admitted were observed on the pediatric floor of a tertiary care children’s hospital. All patients were monitored with continuous pulse oximetry throughout their stay. Any desaturations were documented and treated with repositioning and supplemental oxygen if necessary.

Statistical analysis

Patient charts were reviewed for demographics. This included age, sex, pre-operative AHI, and body mass index (BMI). Other data collected included postoperative saturation of peripheral oxygenation (SpO2), if supplemental oxygen was required, and if patients required an ED or UC visit within 14 days of surgery. Any interventions required within 14 days of discharge were also collected. Patients who were admitted with an AHI of 10 to <20/hr were compared to patients who were discharged with the same AHI range with regard to return to the ED, UC, or other increased medical need after discharge. This was further divided into a group with an AHI of 10 to <15/hr and 15 to <20/hr. For the patients admitted, overnight oxygen desaturations, interventions required, and overall oxygen saturations were recorded and compared for patients with an AHI of 0 to <10/hr, AHI of 10 to <20/hr (subdivided into 10 to <15/hr and 15 to <20/hr), and those with AHI ≥20/hr. Descriptive statistics were given for all data collected. Statistical analysis was performed using Microsoft Excel with the Advanced Statistical Analysis package. Missing data remained missing and were not replaced by substitutions or imputations. Categorical variables are provided as counts and % frequencies. All continuous variables are provided as either means +/- the standard deviation or median and 25th and 75th percentiles, followed by the minimum to maximum dependent on the normality of the data. Univariate analyses of all data were examined between the participants using chi-square tests for categorical variables where appropriate (expected frequency>5 in 80% of cells); otherwise, Fisher’s exact tests were used. Continuous variables were examined using Wilcoxon rank sum tests or t-tests dependent on the normality of the data.

## Results

Demographics

There were 87 patients who met the inclusion criteria. Of these, 46 were male and 41 were female. The age range of these patients spanned from three to 17 years old. The median age was six years. All patients underwent preoperative PSG. Of these patients, two had mild sleep apnea (AHI 1-<5/hr), five had moderate sleep apnea (AHI 5-<10/hr), and 80 patients had severe obstructive sleep apnea (AHI ≥10/hr). Of the patients with severe sleep apnea, 53 patients had an AHI of 10 to <20/hr, and 27 had an AHI of ≥20/hr (Table 1).

**Table 1:** Demographics Values are presented as n (%) for categorical variables and median (range) for continuous variables. AHI: Apnea-hypopnea index

		Group 1: AHI 1-<10/hr	Group 2: AHI 10-<15/hr	Group 3: AHI 15-<20/hr	Group 4: AHI ≥20/hr
Total number of participants	87	7 (8.1%)	37 (42.5%)	16 (18.4%)	27 (31%)
Gender	Male	2	19	5	20
	Female	5	18	11	7
Age (years)	Range	3-7	3-17	4-17	3-17
	Median	3	5	5	7

Mild to moderate obstructive sleep apnea results

There were three patients who had an AHI within the range of mild to moderate OSA (n = 8.1%). Of these patients, two were admitted at the request of the parent. None of the patients required supplemental oxygenation. The average overnight SpO2 was between 93% and 100%, specifically for patients admitted. No patients from this group required an ED or UC visit due to respiratory-related symptoms; however, one patient did require an ED visit three days into the postoperative period for throat pain. The patient was discharged after Motrin administration (Table 2).

**Table 2:** Results for Mild to Moderate and Severe OSA Groups The data has been represented as N (%) of the respective group (1-4). A Fisher exact test was performed to compare the oxygen desaturation results between the groups and resulted in p = 1. A chi-square test was performed to compare ED and UC visits for the four groups, with χ² = 0.41, df = 3, and p = 0.94. A Fisher-Freeman-Halton exact test was run between the four groups and resulted in p = 0.93. Statistical tests could not be run on oxygen requirements overnight between the four groups as there were null (0) results in groups 1, 2, and 3. AHI: Apnea-hypopnea index

	Group 1: AHI 1-<10/hr	Group 2: AHI 10-<15/hr	Group 3: AHI 15-<20/hr	Group 4: AHI ≥20/hr
Number of patients with oxygen desaturation overnight < 94% SpO2	0 (0%)	1 (2.7%)	1 (6.3%)	1 (3.7%)
Required overnight oxygen supplementation	0 (0%)	0 (0%)	0 (0%)	1 (3.7%)
Number of ED or UC visits after discharge	1 (14%)	3 (8.1%)	2 (12.5%)	3 (11%)

Severe obstructive sleep apnea results

There were 53 patients with an AHI of 10 to <20/hr. This was further subdivided, with 37 patients with an AHI of 10 to <15/hr (n = 42.5%), and 16 patients with an AHI of 15 to <20/hr (n = 18.4%). For the AHI group of 10 to <15/hr, 24 of these patients were admitted, and none of the admitted patients required supplemental oxygen. Thirteen of these patients were discharged after surgery. Twenty-three out of the 24 patients admitted had an overnight SpO2 nadir of 94-100%, while one patient had an overnight SpO2 nadir of 85-93%. There were three patients out of the 37 who returned to the ED after discharge; however, only one was for respiratory-related symptoms. This patient initially presented to an UC one day after discharge and was given prednisolone 1 mg/kg and required albuterol every four hours. Thirteen days after surgery, they required admission for an asthma exacerbation. Another patient required fluids for dehydration and an enema for constipation. The third patient required oxycodone for pain management and was discharged from the ED (Table 2). 

The group with an AHI of 15 to <20/hr had nine out of 16 patients admitted postoperatively for overnight observation. None of these patients required supplemental oxygen. The postoperative SpO2 ranged from 94-100% for all admitted patients, with one patient experiencing an oxygen nadir of <94%. Two of these patients required an ED visit after discharge. None of these visits were for respiratory distress (Table 2). 

There were 27 patients with an AHI of ≥20/hr (n = 31%). The group with an AHI of ≥20/hr had 24 out of 27 patients admitted for overnight observation. One patient required supplemental oxygen overnight. Twenty-three out of the 24 patients had an average SpO2 of 94-100% overnight. One patient had a postoperative oxygen nadir of 85-93%. Three of the patients returned to the ED after discharge, and three required further interventions after discharge (Table 2).

Attempts were made to run chi-square tests, Fisher exact tests, and Fisher-Freeman-Halton exact tests. Tests were limited due to low event counts (resulting in multiple categories with a zero value). 

## Discussion

Tonsillectomy is one of the most common pediatric procedures performed, with over 530,000 tonsillectomies being performed in pediatric patients per year [1]. This study examines postoperative respiratory-related events after tonsillectomy for pediatric patients with severe OSA. The severity of OSA was evaluated by using preoperative PSG data. The objective of this study was to evaluate the safety of same-day discharge for pediatric OSA patients with AHI of less than 20/hr. This would expand the current guidelines recommended by the AAO-HNSF, which is to discharge patients with AHI <10/hr [4]. Patients less than three years of age were omitted from the study as there is strong evidence to admit these patients for overnight observation [2,5]. Overall, this study found low respiratory-related complications for all ranges of pediatric OSA patients after T&A. 

Mild to moderate obstructive sleep apnea

Current guidelines state that this group of patients is safe to discharge after surgery, which correlates with our results. Of the patients with mild to moderate OSA, two were admitted, both at parental requests. The two patients did not demonstrate any desaturation events, require supplemental oxygenation, or repositioning. There was one patient from this group with a visit to the emergency department three days after surgery for throat pain and dehydration. This patient has a history of cerebral palsy, contributing to the difficulty of his recovery. He was able to tolerate a soft diet following administration of Motrin and was discharged afterwards. There is limited data for this group due to the majority of patients being discharged. 

Severe obstructive sleep apnea, AHI 10 to <15/hr

Current guidelines by the AAO-HNSF recommend admission for this group of patients; however, no patients in this AHI group who were admitted had overnight desaturations requiring supplemental oxygenation in this study. Also, of the three patients who required an UC or ED visit, only one was for respiratory-related concerns. Of the patients who had an ED visit in this group, one was a visit on postoperative day (POD) 7 for constipation and dehydration. Another patient did present to UC on POD 1 and was given prednisolone 1 mg/kg and required albuterol every four hours. The patient then presented to the ED on POD 13 and was admitted for an asthma exacerbation. Both patients were admitted for overnight observation after surgery and discharged the next morning, without any events overnight. The third patient, who presented to the ED on POD 7, was for dehydration and pain. This patient was not admitted for observation after surgery and did not have any respiratory-related issues. There was one patient from this group who had a postoperative SpO2 reading of 89% overnight. This was resolved with repositioning alone; no supplemental oxygen or further interventions were required. This data supports the safety profile of discharging patients with an AHI <20/hr, as there are minimal respiratory-related events noted. While discharging most patients after T&A with an AHI <20/hr is safe, comorbidities should be considered when deciding whether to admit postoperatively or discharge. Comorbidities such as chromosomal anomalies, neuromuscular disorders, prematurity, obesity, or craniofacial abnormalities are indications to perform tonsillectomy in an inpatient setting. 

Severe obstructive sleep apnea, AHI 15 to <20/hr

None of the patients from this group who were admitted required supplemental oxygenation for desaturations. Of the two patients requiring an ED visit after discharge, neither of these visits was for respiratory-related events. One of these patients presented on POD 4 for nausea, vomiting, and pain. The other patient presented to the ED on POD 1 for blood-tinged sputum. This resolved on its own, and the patient was discharged after several hours of observation. This data supports that patients with an AHI <20/hr can be safely discharged home after T&A, as no respiratory-related events occurred overnight. As no patients within this group had respiratory-related complications, the authors argue that the guidelines can be safely increased to recommend admission for patients with an AHI ≥20/hr and allow patients with an AHI <20/hr with no comorbidities who are stable after surgery to be discharged. 

Severe obstructive sleep apnea, AHI ≥20/hr

Interestingly, three of these patients were not admitted overnight. One patient had an AHI of 25.9/hr. They were eight years old, doing well postoperatively in the PACU, and the decision was made to discharge the patient home. They did not return to the ED or require any further interventions, and no concerns from the parents were noted at the postoperative visit. Another patient who was not admitted had a preoperative AHI of 23.9/hr and was six years old. This patient was also stable in the PACU, and a decision was made to discharge the patient. This patient presented to the ED on POD 5 with a fever of 101.3F and ear pain. They were admitted for dehydration and pain control. The patient was discharged after three days of admission. At the postoperative visit one week later, they were doing well with no further issues. The third patient, who was not admitted, was five years old and had an AHI of 21.1/hr. They did well in the PACU, and it was decided they were stable for discharge. They did not have any respiratory-related events after discharge, require an ED visit, or require further interventions. At their postoperative visit, parents did not express any concerns. 

Of the three patients who returned to the ED after discharge, none were for respiratory-related issues. One of the patients was discussed above. A second patient presented to the ED with ear pain on POD 14 and was diagnosed with left acute otitis media. This patient was admitted initially after surgery and discharged the next day, with an uneventful hospital stay. The third patient presented to the ED on POD 10 for sore throat and dehydration. This patient was 17 years old, had a history of Trisomy 21, and was initially admitted with an uneventful hospital stay. They were seen a month later for their postoperative visit and were doing well with improvement in their sleep apnea.

One patient who was admitted overnight required supplemental oxygen from this group. This patient's SpO2 nadir was 85% and recovered to 94% SpO2 after administration of 1L of oxygen via a nasal cannula. This patient had a preoperative AHI of 46/hr with an oxygen nadir of 70% during their PSG. This is an example that patients with a very high preoperative AHI should be admitted overnight for postoperative monitoring, similar to findings from the current guidelines [4]. The desaturations noted overnight for these patients are often congruent with their preoperative PSG. However, the rare occurrence of the desaturations with even very high AHI in this group provides further support that these pediatric patients are relatively stable postoperatively. This provides further support that discharging patients with an AHI <20/hr is safe. 

Limitations

The population size in this study was limited due to many pediatric patients not acquiring a PSG preoperatively and therefore were omitted from this study. Many physicians state the main reason they order a preoperative PSG in pediatric patients is discordance between tonsil size and symptom severity. When patients present with clear symptoms and tonsillar hypertrophy, the most common cause of pediatric OSA, the PSG is often forgone [5]. Another reason PSG is not always acquired is that waiting for a PSG will delay the treatment of OSA. The average wait time for a pediatric PSG appointment in the surrounding areas where the study was performed is three months. The risks of untreated OSA may outweigh the benefit of obtaining a definitive diagnosis of OSA in the pediatric patient. Untreated OSA is associated with cardiovascular disease, hypertension, metabolic disease, decreased problem-solving and attention, depression, and decreased school function [6,7]. Additionally, limitations include the retrospective design, single-center setting focus, and surgeon selection preference for patient admission vs discharge. 

Future directions

Future studies may benefit from a larger, prospective multicenter study. This can be accomplished by making pediatric PSG more widely available or considering reliable at-home sleep studies to be able to diagnose pediatric patients with OSA without the risk of longer duration of untreated sleep apnea. 

Additional research may also be conducted on the subgroups of patients with an AHI ≥20/hr to determine if it is safe to discharge pediatric patients with an AHI between 20 and 25/hr.

## Conclusions

This study underscores the significance of adenotonsillectomy as a procedure widely used to treat OSA and its safety profile. Consistent with current evidence, this paper suggests the safe discharge of pediatric tonsillectomy patients who have an AHI less than 20. However, prospective multicenter studies are needed to validate these findings before broad changes to current clinical practice guidelines can be recommended. If confirmed, adoption of a higher AHI threshold for postoperative admission could reduce unnecessary overnight hospitalizations, decrease healthcare expenditures, optimize inpatient bed utilization, and allow patients and their families to return home sooner without compromising patient safety.
